# Systematic review of cochlear implantation in patients with inner ear malformations

**DOI:** 10.1371/journal.pone.0275543

**Published:** 2022-10-21

**Authors:** Sunny Shah, Rameen Walters, Jake Langlie, Camron Davies, Ariel Finberg, Maria-Pia Tuset, Dario Ebode, Rahul Mittal, Adrien A. Eshraghi

**Affiliations:** 1 Hearing Research and Cochlear Implant Laboratory, Department of Otolaryngology, University of Miami Miller School of Medicine, Miami, Florida, United States of America; 2 Herbert Wertheim College of Medicine, Florida International University, Miami, Florida, United States of America; 3 Department of Neurological Surgery, University of Miami Miller School of Medicine, Miami, Florida, United States of America; 4 Department of Biomedical Engineering, University of Miami, Coral Gables, Florida, United States of America; 5 Department of Pediatrics, University of Miami Miller School of Medicine, Miami, Florida, United States of America; Universidade Federal de Sao Paulo/Escola Paulista de Medicina (Unifesp/epm), BRAZIL

## Abstract

**Objectives:**

To evaluate the outcomes of cochlear implantation in patients with severe to profound sensorineural hearing loss due to inner ear malformations (IEMs) when compared to patients without IEMs. We discussed audiological outcomes such as open-set testing, closed-set testing, CAP score, and SIR score as well as postoperative outcomes such as cerebrospinal fluid gusher and incomplete insertion rate associated with cochlear implantation in individuals with IEMs.

**Data sources:**

PubMed, Science Direct, Web of Science, Scopus, and EMBASE databases.

**Review methods:**

After screening a total of 222 studies, twelve eligible original articles were included in the review to analyze the speech and hearing outcomes of implanted patients with IEMs. Five reviewers independently screened, selected, and extracted data. The “Tool to Assess Risk of Bias in Cohort Studies” published by the CLARITY group was used to perform quality assessment on eligible studies. Systematic review registration number: CRD42021237489.

**Results:**

IEMs are more likely to be associated with abnormal position of the facial nerve, raising the risk of intraoperative complications. These patients may benefit from cochlear implantation, but audiological outcomes may also be less favorable than in individuals without IEMs. Furthermore, due to the risk of cerebrospinal fluid gusher, incomplete insertion of electrodes, and postoperative facial nerve stimulation, surgeons can employ precautionary measures such as preoperative imaging and proper counseling. Postoperative imaging is suggested to be beneficial in ensuring proper electrode placement.

**Conclusions:**

Cochlear implants (CIs) have the potential to provide auditory rehabilitation to individuals with IEMs. Precise classification of the malformation, preoperative imaging and anatomical mapping, appropriate electrode selection, intra-operative techniques, and postoperative imaging are recommended in this population.

## Introduction

The indications for cochlear implantation are continually expanding beyond severe-to-profound deafness to include patients with single-sided deafness and, more recently, congenital anatomical variations of the inner ear [1–5]. When considering congenital cases of sensorineural hearing loss (SNHL), inner ear malformations (IEMs) account for roughly 20% of SNHL cases, with 20–35% of those meeting criteria for cochlear implantation [6, 7].

Due to the increased use of magnetic resonance imaging (MRI) and computerized tomography (CT) temporal bone scanning, otologists are starting to recognize and identify more IEMs [8]. As the cochlear implant (CI) recipient population continues to include more patients with IEM, there is a need to retrospectively review the clinical and audiological outcomes among this population. When reviewing patients with IEM undergoing cochlear implantation, consideration of both surgical outcomes, evaluating the safety of CI among this patient subset, and audiological outcomes, indicating recovery of speech and auditory perception, can provide insight into the efficacy of cochlear implantation [9]. Given the variable anatomy of patients with IEM, careful planning and consideration before cochlear implantation should include identifying electrode subtype, determining length and depth of insertion, and deciding between CI or auditory brainstem implantation (ABI) to tailor treatment to the presented malformation [6, 10, 11].

The objective of this systematic review article is to analyze current literature to evaluate outcomes of cochlear implantation in treating SNHL in patients with congenital IEMs, when compared to patients without IEMs. We discussed open-set testing, closed-set testing, CAP score, SIR score, and surgical complications such as cerebrospinal fluid gusher and incomplete insertion rate associated with cochlear implantation in individuals with IEMs. This review hopes to guide the management of perioperative and postoperative complications that arise in patients with IEMs as well as discuss, stratify, and predict audiological as well as surgical outcomes of patients based on the IEM subtype.

## Methods

### Search strategy

This study was conducted following the Preferred Reporting Items for Systematic Reviews and Meta-Analyses (PRISMA) statement and supplemented by guidance from the Cochrane Collaboration Handbook. A protocol of this systematic review was designed *a priori* and was registered in the PROSPERO database (registration number: CRD42021237489) Searches were performed from January 1, 2016 until February 14, 2022 in the following databases: PubMed (MEDLINE), Science Direct, Web of Science, Scopus, and EMBASE databases using the following MeSH terms: ("Cochlear Implants"[Mesh] OR "Cochlear Implantation"[Mesh]) AND ("Ear, Inner/abnormalities"[Mesh] OR "Ear, Inner/growth and development"[Mesh]), where MeSH search was not available the following Boolean terms were used ("Cochlear Implant" OR "Cochlear Implantation") AND ("Inner ear abnormalities" OR "cochlear abnormalities" OR "Congenital malformation").

### Study selection

Studies were excluded based on the following exclusion criteria: studies that did not fit the above characteristics, review articles, meta-analyses, abstracts only, conference proceedings, editorials/letters, case reports, or articles published before 2016. Articles prior to 2016 were excluded as they were previously evaluated in a systematic review [4]. Additionally, adult patients were excluded from analysis. The following inclusion criteria were used: data including closed-set word (CSW) tests, open-set word (OSW) tests, surgical data including intraoperative and postoperative complications, Categories of Auditory Performance (CAP), and/or Speech Intelligibility Rating (SIR) in patients undergoing unilateral or bilateral cochlear implantation for SNHL secondary to IEMs. All searched titles, abstracts, and full-text articles were independently reviewed by at least two reviewers (S.S., R.W., J.L., A.F., and C.D.). Disagreements over inclusion and exclusion criteria were resolved through a consensus between the reviewers or discussion with other investigators of this study. Articles were initially screened based on title and abstract screening before proceeding to full-text analysis.

### Quality assessment

The “Tool to Assess Risk of Bias in Cohort Studies” published by the CLARITY group was used to perform quality assessment of the studies [12]. Eight areas were evaluated: population selection, exposure assessment, pre-existing exposure, control matching, confounding assessment, assessment of outcome, follow-up assessment, and co-intervention assessment. The risk of bias was rated as low, unclear, or high according to established Cochrane guidelines. At least two reviewers independently conducted this assessment (S.S., R.W., J.L., A.F., and C.D.), and any disagreements were resolved by consensus between the reviewers or discussion with other investigators of this study.

### Data extraction

All data were extracted and separated by type of malformation [6, 7]. These malformations included: Michel deformity, common cavity (CC) deformity, cochlear aplasia, cochlear dysplasia, cochlear hypoplasia, enlarged vestibular aqueduct (EVA) syndrome, incomplete partition types I (IP-I), II (IP-II or Mondini deformity), and III (IP-III). Descriptions and audiological findings of these malformations are included in Table 1 [13]. Any discrepancies in classification or grouping were discussed with the senior author (A.E.) for a final decision.

**Table 1:** Descriptions and audiological findings in individuals with IEMs [13].

IEM	Description	Audiological Findings
Common Cavity Deformity	Vestibule and cochlea are combined into one round structure	Profound sensorineural hearing loss
Michel Deformity	Complete absence of cochlea, vestibule, semicircular canals, vestibular and cochlear aqueducts	Profound sensorineural hearing loss
Cochlear Aplasia	Absence of a cochlea. Multiple forms with normal labyrinth or dilated vestibule	Profound sensorineural hearing loss
Hypoplastic Cochlea	Cochlea and vestibule are clearly separated but cochlea’s external dimensions are smaller than normal. Multiple forms of CH-I, CH-II, CH-III, and CH-IV	Partial sensorineural, conductive, or mixed hearing loss
Enlarged Vestibular Aqueduct Syndrome	Normal cochlea, vestibule, and semicircular canals with a vestibular aqueduct that is larger than 1.5mm	Spectrum of normal hearing to profound mixed or sensorineural hearing loss that may be progressive in nature
Incomplete Partition Type I	Cochlea and vestibule are clearly separated but lacks the modiolus and interscalar septa	Profound sensorineural hearing loss
Incomplete Partition Type II	Cochlea and vestibule are clearly separated but the apical portion of modiolus is malformed	Spectrum of normal hearing to profound mixed or sensorineural hearing loss that may be progressive in nature
Incomplete Partition Type III	The internal acoustic meatus is wide and directly opens up to the basal turn of the cochlea.	Mixed or sensorineural hearing loss

Intraoperative and perioperative complications were grouped into cerebrospinal fluid (CSF) leak or gusher, postoperative infections, electrode-induced facial nerve stimulation, and complete versus incomplete electrode insertion.

Speech perception tests were grouped into CSW or OSW tests depending on which category they fell into. Furthermore, data regarding speech perception abilities were extracted from multiple studies that included SIR scores. Finally, CAP results were also included to stratify auditory receptive skills apart from speech perception in patients following cochlear implantation.

## Results

**Fig 1:**
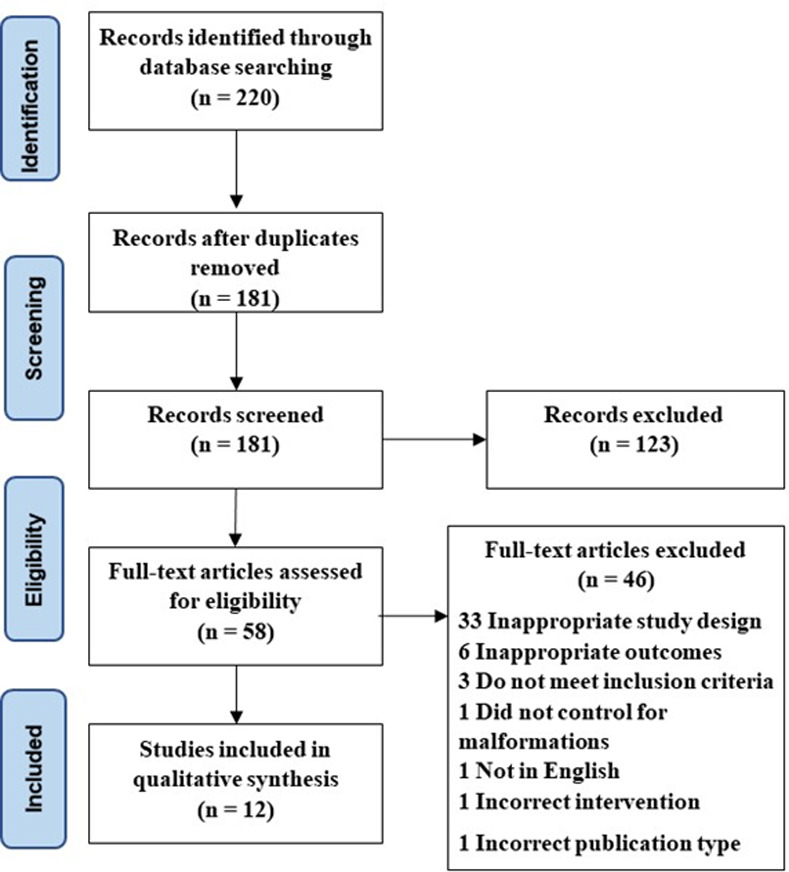
Preferred reporting items for systematic reviews and meta-analyses (PRISMA) flowchart detailing study design.

A total of 220 studies were retrieved using the predefined search algorithm. After deduplication, 181 studies were included for title and abstract screening. After screening, 123 studies were excluded based on irrelevance and 58 articles were included for whole-text analysis. A total of 46 articles were then excluded as 3 had the inappropriate study design, six measured inapplicable outcomes, three studied the inappropriate patient population, one did not control for malformations, one was not published in English, one had the incorrect intervention, and one was the incorrect publication type. Finally, 12 articles remained for inclusion in the literature review and qualitative analysis (Fig 1).

Twelve articles published between the years of 2016–2021 were included in this systematic review. A total of 6,262 patients were evaluated across the 12 articles included. Baseline characteristics are outlined in Table 2. Risk of Bias is outlined in Table 3. Out of these studies, speech perception test results were included in four studies, operative findings were included in six studies, CAP was included in two studies, and SIR was included in three studies. Most of the studies (7/12) were retrospective cohort studies where the exposure group was patients with IEMs and the control group was patients with normal inner ear anatomy (Table 4). The search strategy employed for the studies included is shown in the PRISMA diagram in Fig 1. A summary of each study design, patient grouping, and included results is presented in Table 4.

**Table 2:** Patient characteristics.

	Celik, 2018	Choi, 2016	Isaiah, 2016	Kamogashira, 2016	Melo, 2017	Ozkan, 2020	Parent, 2018	Patel, 2018	Qi, 2018	Ronner, 2020	Vashist, 2016	Zhang, 2017
Patients with IEM	28	8	102	20	26	137	136	18	108	47	17	6
Patients without IEM	41	80	N/A	20	303	137	4194	N/A	592	107	129	6
Age at CI Surgery—Patients with IEM*	12.6 +/- 3.9 months	95.5 +/- 114.6 months	32.5 +/- 18.8 months	32.4 +/- 13.2 months	25.5 +/- 5.5 months	36.9 +/- 21.3 months	N/A	23.8 +/- 17.0 months	19.2 +/- 7.7 months	46.3 months (0–180)	N/A	76.5 +/- 60.0 months
Age at CI Surgery—Patients without IEM*	14.2 +/- 4.2 months	93.1 +/- 111.6 months	N/A	30.0 +/- 8.4 months	43 months (12–94)	38.2 +/- 20.5 months	N/A	N/A	18.9 +/- 7.6 months	33.6 months (0–180)	N/A	72.8 months
Gender—Patients with IEM	16 males, 12 females	11 males, 0 females	Not Available	10 males, 10 females	N/A	64 males, 73 females	N/A	8 males, 10 females	362 males, 230 females	25 males, 22 females	N/A	5 males, 1 female
Gender—Patients without IEM	23 males, 18 females	N/A	N/A	10 males, 10 females	N/A	61 males, 76 females	N/A	N/A	57 males, 51 females	55 males, 52 females	N/A	5 males, 1 female
Total Implanted IEM Ears (Percent of IEM in Study)	28	11	134	23	26	137	136	18	108	79	17	6
*Cochlear Aplasia*	-	-	3 (2%)	-	-	-	-	-	-	-	-	-
*Cochlear Hypoplasia*	8 (29%)	-	-	8 (35%)	9 (35%)	26 (19%)	-	-	-	6 (8%)	-	-
*Cochlear Dysplasia*	8 (29%)	-	40 (30%)	8 (35%)	9 (35%)	26 (19%)	-	-	-	9 (12%)	-	-
*Incomplete Partition Type-1*	5 (18%)	-	-	5 (21%)	-	36 (26%)	-	-	-	-	-	-
*Mondini Deformity / Incomplete Partition Type-2*	7 (25%)	-	-	2 (9%)	5 (19%)	40 (29%)	-	12 (67%)	108 (100%)	12 (15%)	6 (35%)	-
*Incomplete Partition Type -3*	-	11 (100%)	-	-	-	10 (7%)	-	-	-	-	-	-
*Common Cavity*	2 (7%)	-	-	2 (9%)	-	8 (6%)	-	-	-	2 (2%)	-	6 (100%)
*Complete Labyrinthine Aplasia (Michel Deformity)*	2 (7%)	-	-	-	-	-	-	-	-	-	-	-
*Vestibular Dysplasia*	-	-	24 (18%)	-	-	-	-	-	-	-	-	-
*Enlarged Vestibular Aqueduct*	-	-	23 (17%)	-	6 (23%)	17 (13%)	-	6 (33%)	-	22 (28%)	3 (18%)	-
*Semicircular Canal Dysplasia/Aplasia*	-	-	-	2 (9%)	5 (19%)	-	-	-	-	15 (19%)	7 (41%)	-
*Cochlear Aperture Abnormalities*	-	-	4 (3%)	1 (4%)	-	-	-	-	-	3 (4%)	-	-
*Other* **	4 (14%)	-	40 (30%)	-	1 (4%)	-	-	-	-	16 (20%)	1 (6%)	-
*Unspecified*	-	-	-	3 (13%)	-	-	136 (100%)	-	-	-	-	-

*Mean plus/minus standard deviation given when available; when not, age range at time of implantation is given in months in parenthesis

**Other includes cochlear nerve aplasia (16), cochlear nerve hypoplasia (19), dysplastic stapes (1), labyrinthitis ossificans (23), and vestibular nerve hypoplasia (3)

**Table 3:** The results of risk of bias analysis for the 12 studies examined in this review using the Cochrane cohort study risk of bias tool.

Author (Year)	Study Type	Population Selection	Exposure Assessment	Pre-existing Exposure	Control Matching	Confounding Assessment	Assessment of Outcome	Follow-up Assessment	Co-intervention Assessment	Score (Lower is Better)
**Celik (2018)**	**Prospective Cohort**	**DY**	**DY**	**DY**	**DN**	**DN**	**PY**	**PY**	**PY**	**9**
**Choi (2016)**	**Retrospective Cohort**	**PN**	**DY**	**DY**	**PY**	**PY**	**DY**	**DY**	**PY**	**5**
**Isaiah (2017)**	**Retrospective Cohort**	**PN**	**DY**	**DY**	**DN**	**DY**	**DY**	**PY**	**PN**	**8**
**Kamogashira (2016)**	**Retrospective Cohort**	**PN**	**DY**	**DY**	**PY**	**PN**	**DY**	**PY**	**PN**	**8**
**Melo (2017)**	**Retrospective Cohort**	**PN**	**DY**	**DY**	**PY**	**PN**	**DY**	**PN**	**PN**	**9**
**Ozkan (2021)**	**Prospective Cohort**	**DY**	**DY**	**DY**	**PN**	**DY**	**PY**	**PY**	**PY**	**5**
**Parent (2020)**	**Prospective Cohort**	**DY**	**DY**	**DY**	**DY**	**DY**	**DY**	**DN**	**DN**	**6**
**Patel (2018)**	**Retrospective Cohort**	**PN**	**DY**	**PY**	**PN**	**PN**	**DY**	**PY**	**PN**	**10**
**Qi (2019)**	**Prospective Cohort**	**DY**	**DY**	**DY**	**PN**	**PY**	**DY**	**DY**	**DY**	**3**
**Ronner (2020)**	**Retrospective Cohort**	**PN**	**DY**	**DY**	**DN**	**PN**	**DY**	**PY**	**PY**	**9**
**Vashist (2016)**	**Prospective cohort**	**PN**	**DY**	**DY**	**DN**	**DN**	**DY**	**PY**	**DN**	**12**
**Zhang (2017)**	**Retrospective Cohort**	**PN**	**PY**	**DY**	**PN**	**PY**	**DY**	**DY**	**DY**	**6**
**Key**			**DY** **Definitely Yes**		**PY** **Probably Yes**		**PN** **Probably No**		**DN** **Definitely No**	

Overall scores were assigned to each paper with the following rubric and cutoffs: Definitely Yes: 0, Probably Yes: 1, Probably No: 2, Definitely No: 3; Scores between 0–5 Green, 6–8 Yellow, 9–11 Orange, >11 Red. Higher scores indicate a higher risk of bias.

**Table 4:** Characteristics of all included studies.

Reference	Sample Size	Surgical Complications	Audiometric Outcomes
**Celik (2018)**	69 atients • Group 1: 41 normal anatomy • Group 2: 28 IEM	N/A	MTP Scores: Group 1 • 3-word = 7.5(62%) • 6-word = 10.4(58%) • 12-word = 14(59%) Group 2 • 3-word = 7.7(64%) • 6-word = 10.6(59%) • 12-word = 14(59%) No statistically significant difference between the two groups
**Choi (2016)**	88 atients • Group 1: 80 normal anatomy • Group 2: 8 IP-III	Intraoperative: CSF Gusher occurred in all 8 IP-III patients Postoperative: No postoperative complications	CAP Scores: Group 1 • 3 month = 2.8 • 6 month = 3.5 • 12 month = 4.1 • 18 month = 4.3 • 24 month = 4.8 Group 2 • 6 month = 3.0 • 12 month = 3.6 • 24 month = 3.9 *No statistically significant difference in CAP score between Groups 1 & 2
**Isaiah (2017)**	381 patients • Group 1: 279 normal anatomy • Group 2: 102 IEM	Intraoperative: CSF Gusher occurred in 5 patients Incomplete Insertion in 5 patients	Speech Perception Group 1 • 70% achieved open or closed speech discrimination Group 2 • Cochlear dysplasia 76% failed both open and closed speech discrimination. • Vestibular Dysplasia 88% failed both open and closed speech discrimination. • EVA/Dilated ES 24% failed both open and closed speech discrimination. • Cochlear Nerve Hypoplasia 100% failed both open and closed speech discrimination.
**Kamogashira (2016)**	40 patients • Group 1: 20 normal anatomy • Group 2: 20 IEM	Intraoperative: CSF Gusher occurred in 5 patients Postoperative: Facial Nerve Stimulation occurred in 5 patients	N/A
**Melo (2017)**	329 patients • Group 1: 303 normal anatomy • Group 2: 26 IEM	Intraoperative: Incomplete insertion in 3.8% of patients with Cochlear hypoplasia CSF gusher in 7.6% of patients with IP-II	Speech Perception: Group 1 • Monosyllabic test = 69.6% • Number test = 99.9% • Sentences test = 69.5% Group 2 • Monosyllabic test = 78.1% • Number test = 100% • Sentences test = 78.9% CAP Score: • Group 1 = 6.9 • Group 2 = 7.1 SIR Score: • Group 1 = 4.3 • Group 2 = 4.3 *No statistically significant difference for CAP and SIR scores
**Ozkan (2021)**	274 patients • Group 1: 137 normal anatomy • Group 2: 137 IEM	N/A	Speech Perception: Group 1 • Closed Set Pattern Perception Testing at 1–3 years = 78%-93% • Open Set Sentence Recognition Testing at 1–3 years = 42%-87% Group 2: • Closed Set Pattern Perception Testing at 1–3 years • Enlarged Vestibular Aqueduct = 90% • IP-II = 88% • Dilatation of Vestibule = 56% • IP-III = 50% • IP-I = 74% • Cochlear Hypoplasia = 48% • Common Cavity = 35% • Open Set Sentence Recognition Testing at 1–3 years • Enlarged Vestibular Aqueduct = 80% • IP-II = 48% • Dilatation of Vestibule = 56% • IP-III = 34% • IP-I = 12% • Cochlear Hypoplasia = 6% • Common Cavity = N/A (Did not qualify)
**Parent (2020)**	5728 patients • Group 1 = 4194 unknown etiology of hearing loss • Group 2 = 168 IEMs	Operative Complications in Group 1 occurred in 245 patients Operative Complications in Group 2 occurred in 17 patients	N/A
**Patel (2018)**	18 = Ears • Group 1 = 6 normal anatomy • Group 2 = 12 incomplete partition	N/A	Word Recognition Score: IP-II patients experienced a non-statistically significant difference of 8% in WRS when compared to controls.
**Qi (2019)**	700 patients • Group 1 = 592 normal anatomy • Group 2 = 108 mondini dysplasia	N/A	SIR Score: Statistical differences at same time point Group 1 • Preoperative = 0.306 • 1 month = 0.697 • 3 month = 0.505 • 6 month = 0.246 • 12 month = 0.203 • 24 month = 0.352 • 36 month = 0.752 • 48 month = 0.251 • 60 month = 0.568 Group 2 • Preoperative = 0.124 • • 1 month = 0.283 • 3 month = 0.361 • 6 month = 0.531 • 12 month = 0.204 • 24 month = 0.722 • 36 month = 0.523 • 48 month = 0.628 • 60 month = 0.858
**Ronner (2020)**	154 ears • Group 1: 107 normal anatomy • Group 2a: 31 Low Risk Anatomy (Enlarged Vestibular Aqueduct, Lateral Semicircular Canal Abnormalities, IP-II, and Wide Internal Auditory Canal) • Group 2b: 16 High Risk Anatomy (Cochlear Dysplasia, Cochlear Aperture Abnormalities, Common Cavity Deformity, and Cochlear Nerve Deficiency)	N/A	Group 1 • *Preoperative Score (percentage): 1 patient (60–69), 1 patient (50–59), 1 patient (40–49), 1 patient (30–39), 3 patients (20–29), 1 patient (10–19), 1 patient (0–9) • *Postoperative Score: 15 patients (90–100), 16 patients (80–89), 16 patients (70–79), 2 patients (60–69), 1 patient (30–39), 1 patient (20–29), 1 patient (10–19) Group 2a • *Preoperative Score: 1 patient (60–69), 1 patient (10–19) • *Postoperative Score: 2 patients (90–100), 4 patients (80–89), 6 patients (70–79), 1 patient (60–69), 2 patients (50–59), 1 patient (40–49), 2 patients (30–39), 1 patient (20–29), 1 patient (0–9) Group 2b • *Preoperative Score: 1 patient (10–19), 1 patient (0–9) • *Postoperative Score: 5 patients (90–100), 3 patients (70–79), 2 patients (60–69),1 patient (20–29) *Speech perception outcomes were not found for all patients undergoing cochlear implantation
**Vashist (2016)**	146 patients • Group 1: 131 normal anatomy • Group 2: 17 IEM	4 patients with normal anatomy experienced CSF leakage 5 patients with IEM experienced CSF leakage	Group 1 • Average CAP Score of patients who experienced CSF leakage = 4.5 Group 2 • Average CAP Score of patients who experienced CSF leakage = 4.5
**Zhang (2017)**	12 patients • Group 1: 6 normal anatomy • Group 2: 6 IEM	N/A	Group 1 CAP and SIR scores not specifically included for control patients Group 2 Preoperative CAP Score • Patient 1 = 0 • Patient 2 = 0 • Patient 3 = 0 • Patient 4 = 3 • Patient 5 = 0 • Patient 6 = 2 Postoperative CAP Score • Patient 1 = 2 • Patient 2 = 3 • Patient 3 = 3 • Patient 4 = 4 • Patient 5 = 4 • Patient 6 = 4 Preoperative SIR Score • Patient 1 = 1 • Patient 2 = 1 • Patient 3 = 1 • Patient 4 = 1 • Patient 5 = 1 • Patient 6 = 1 Postoperative SIR Score • Patient 1 = 1 • Patient 2 = 1 • Patient 3 = 2 • Patient 4 = 2 • Patient 5 = 2 • Patient 6 = 2

### Open-set testing

Overall, three studies included open-set results. Isaiah et al. included 78 patients with IEMs. One year after CI surgery, results showed that 33% of control patients failed open-set speech discrimination. Multiple groups with IEMs failed (defined by a total score of 0%) in both open- and closed-set speech testing. This included 77% with cochlear dysplasia and 89% with vestibular dysplasia [14]. Differences between the success rates of speech performance testing between these IEM groups were not statistically significant [14].

In the study of Melo et al., patients were assessed once between 8–180 months post-implantation. Although not statistically significant, the average test scores on monosyllabic word sets in patients with IEMs was 78.1% compared to 69.6% in the controls [15].

In Ozkan et al., open-set sentence recognition testing was performed [16]. At the 1–3-year mark, IEM patients without common cavity deformity had a maximum score of 80%, while IEM patients with common cavity did not score high enough on the pattern perception test for sentence recognition testing.

### Closed-set testing

Five studies with closed-set speech perception tests were included in the final dataset. In the study of Celik et al., control patients did not have congenital IEMs. IEMs in the study included cochlear hypoplasia, IP-II, IP-I, and CC. Both groups scored similarly on the 6-month postoperative Monosyllabic-Trochee-Polysyllabic Test (MTP) in the 3-word groups (Control = 7.5, IEMs = 7.7), 6-word groups (Control = 10.4, IEMs = 7.7), and 12-word groups categories (Control = 14.3, IEMs = 14.0) [17]. The results of this study were not statistically significant.

Isaiah et al. evaluated the CSW ability of their participants using the Early Speech Perception (ESP) test. In patients with IEMs, 9% of those with cochlear dysplasia achieved a non-zero score on the closed-set evaluation alone, along with 6% of patients with vestibular dysplasia and 10% with EVA syndrome [14].

Ronner et al. discussed the preoperative and postoperative early speech perception word intelligibility scores for cochlear implantation patients. Patients were stratified into normal anatomy, low-risk anatomy, and high-risk anatomy (Table 4) [18].

In the study of Ozkan et al., closed-set pattern perception testing was done on both control and experimental patients. Patients with IEMs were compared against one another; those with dilatation of the vestibule, IP-I, cochlear hypoplasia, and CC deformity performed statistically significantly worse when compared to patients with EVA, IP-II, and IP-III. At the 1-3-year postoperative time point, EVA patients scored 90% on pattern perception test, while patients with CC deformity scored approximately 35%.

Patel et al. found that when comparing patients with IP-II to patients without IP-II undergoing cochlear implantation, patients with IP-II had a statistically significant decrease of 30.2% in word recognition score (WRS) following cochlear implantation to patients with normal anatomy [19].

### CAP score

Three studies included a preoperative and postoperative CAP score. Zhang et al. evaluated children with CC deformity against a control group matched in sex, age, and time of implantation (n = 6) [20]. Both the control and IEM groups showed improvement, though patients with CC deformity improved significantly less than those with normal inner ear anatomy. The mean CAP improvement in patients with IEMs was 3.50 compared to 9.50 in the control group [20].

Melo et al. assessed CAP-II scores in patients with and without IEMs that received CIs. The authors defined major IEMs as cochlear hypoplasia and IP-II, while all other anomalies were considered minor IEMs [15]. These values were not statistically significant, and there was no statistically significant difference between the patients with IEMs and those in the control group at 12, 24, and 36 months post-cochlear implantation [15].

In Choi et al., CAP scores were measured in patients with IP-III deformity and in control patients with normal inner ear structure before and after surgery. Audiologic assessments occurred pre-cochlear implantation and at 3, 6, 12, 18, and 24 months after implantation, and the mean CAP scores in the IP-III patients were 0.8, 2.6, 3.0, 3.6, and 3.9, respectively [21]. When compared to the control groups, the scores were not statistically significant at any time point before 24 months postoperatively. At 24 months, the patients with IP-III deformity performed significantly poorer than the controls with a mean CAP of 3.9 versus 5.0.

### SIR score

Three studies included SIR scores as an outcome measure. Zhang et al. evaluated SIR scores in patients with CC deformity compared with a sex, age, and time of implantation matched control group. In congruity with their CAP score outcomes, both the CC and normal anatomy groups demonstrated improvement following cochlear implantation; however, those with CC deformity showed significantly less improvement in SIR 1-year postoperatively. The mean SIR score in the common cavity group was 3.75, while the control group scored 9.25 [20].

Melo et al. also included SIR scores as post-cochlear implantation audiometric outcomes. Mean SIR score 24 months following surgery in those with major IEMs was 3.71 (+/-1.43), while those with minor IEMs was 4.41 (+/-0.90) [15]. Both groups had an average SIR score of 4.3 with a variability of +/-1.0 in patients without IEMs versus a +/-1.1 in patients with IEMs [15]. Average SIR score in patients with IEMs were lower when compared to the controls at 12-, 24-, and 36-months following surgery; however, these differences were not statistically significant [15].

Qi et al. conducted a study evaluating SIR scores in patients for 5 years with rating assessments occurring before surgery and at 1, 3, 6, 12, 24, 36, 48, and 60 months postoperatively. Both groups demonstrated significant improvements between each of the time points after 3 months except for the 6–12-month period and the 36–48-month period; no statistically significant difference in SIR was found at these time points [22].

### Operative findings

Operative findings were included in a total of five studies within this review. In all patients in Kamogashira et al., the standard facial recess approach was adopted, and a CI electrode was inserted through a cochleostomy. Intraoperative CSF gushers were encountered in 38% of children with cochlear hypoplasia and 29% with IP. No patients experienced facial nerve palsy; however, postoperative facial nerve stimulation by CI was frequently observed in children with CC (50%) and cochlear hypoplasia (38%) [23].

In Vashist et al., 29% of patients with IEMs experienced CSF gushers (2 EVA and 2 IP-II) or CSF oozing (1 IP-II) while only 3% with normal inner ear anatomy suffered from CSF gushers [24]. Only patients with IP-I, IP-II, IP-III, EVA syndrome, dysplastic semicircular canal, and narrow internal auditory canal (IAC) were included. CSF oozing was defined as spontaneous resolution of CSF leakage within 5–10 minutes, while CSF gusher was defined as resolution of leakage within 15–20 minutes only after packing of the round window site [24]. All 9 patients with CSF leakage had complete insertion of the electrode, and there were no cases of CSF otorhinorrhea or meningitis in the follow-up period.

In Isaiah et al., a total of 5 patients (6.4%) had an intraoperative complication of CSF gusher; 4 of these patients had associated cochlear dysplasia alone or in conjunction with another IEM. In addition, 6.4% of patients had incomplete insertion of the electrode (3 cochlear dysplasia), while 3.8% had full insertion with difficulty [14].

In Melo et al. 9 patients had cochlear hypoplasia, 5 had IP-II, 3 had EVA syndrome, 3 had EVA syndrome with semicircular canal (SCC) aplasia, 5 had partial SCC aplasia, and 1 patient had cochlear nerve hypoplasia [15]. Only 3.8% of patients with cochlear hypoplasia had surgery complicated by incomplete electrode insertion; all other patients had successful complete insertion of the electrodes. CSF leakage occurred in 7.6% of patients, all of which had IP-II. The study did not assess postoperative facial nerve stimulation.

In Choi et al., all 8 patients with IP-III experienced a CSF gusher at the cochleostomy site during the procedure [21].

In Parent et al., patients with cochlear malformations suffered from an increased rate of statistically significant complications. Multivariate analysis found that adult patients with cochlear malformations had a higher rate of complications than pediatric patients [25]. The combined minor and major complication rate was 10.12% (6.4%-15.6%) in patients suffering from cochlear malformations; however, the type of complication was not stratified by etiology of deafness [25].

## Discussion

The findings from 12 studies included in this systematic review article suggest that patients with IEMs can improve in both speech perception and speech production with little intra- and postoperative risk. As these improvements can vary, pre-implantation conversations with parents should emphasize appropriate expectations following implantation. Risk of Bias analysis indicated that most studies had a moderate risk of bias, as noted by an overall bias score between 6–10 calculated by addition of each Risk of Bias component. OSW testing indicated no statistically significant difference in speech perception between patients with or without IEMs. In contrast, CSW testing showed that patients with more severe IEMs, some of which affect the cochlear nerve, may perform worse when compared to control patients. Similarly, patients with CC deformity or IP-III malformations performed worse on CAP testing when compared to control patients. However, SIR scores did not produce a statistically significant difference between the control and IEM groups. As expected, due to anatomical configurations, CSF gushers occurred more frequently in patients suffering from EVA syndrome, IP I-III, and cochlear dysplasia. Postoperative facial nerve stimulation occurred more frequently in CC or cochlear hypoplasia.

Farhood et al. concluded that cochlear implantation can still be beneficial in most patients with IEMs if patients’ expectations are managed and operative risks are adequately discussed [4]. Patients with IEMs have significant improvement in speech perception and audiological outcomes, with minimal improvement observed within the first year. Equivalent audiometric outcomes were seen in all patients with IEMs; however, care should be taken when implanting patients with EVA syndrome, IP I-III, cochlear hypoplasia, and CC deformity due to increased risk of intraoperative and postoperative complications. Despite these potential complications, we found that early implantation appears to yield satisfactory audiometric outcomes in all patients suffering from congenital IEMs. Therefore, we recommend implantation as indicated, in these populations, albeit with special precautions for risk prone IEM subsets.

### Speech perception results

Studies suggest that patients with IEMs take longer to develop speech perception skills than those without IEMs [14, 15, 17–19]. However, as time of post-implantation progresses, patients with IEMs show no statistically significant difference in speech perception on open-set tests compared to children with normal cochlear anatomy. Although most IEM patients fare well in speech perception 2–3 years after implantation, IEMs such as common cavity deformity or severe cochlear hypoplasia that disrupt cochlear architecture or cochlear nerve formation have limited improvement and tend to perform much worse in all speech perception tests [26, 27]. Patients with milder forms of cochlear hypoplasia tend to perform better. Many studies suggest that earlier age of implantation resulted in better performance on open-set word speech perception tests [28, 29]. Similar to their performance on the open speech perception test, IEM patients performed comparably to controls in the closed speech perception test at multiple time points post-implantation, including at 6, 12, and 24 months [21].

### Auditory outcomes

The three studies that included a CAP score indicated its usefulness in determining auditory perception beyond speech perception. This is important given that children with IEMs may have more difficulty understanding speech than environmental sounds. In all studies analyzed, patients with CC deformity performed the worst in CAP scores, likely due to concurrent malformation of the cochlear nerve. Although CC deformity patients did not perform well, they still experienced improvement compared to preoperative CAP scores, as did all patients with other IEMs. Most studies showed that, in patients with IP-II and IP-III malformations, there were no statistically significant differences in CAP score when compared to normal anatomy controls. Choi et al. showed that improvements in CAP score in patients with IP-III performance halted at 24-months following cochlear implantation and that they scored worse than control patients. However, Daneshi et al. emphasizes that CAP score results are subjective and commonly rely on parental statements and observations rather than objective measures [30].

The speech perception was analyzed in three studies using the SIR score. In conjunction with CAP scores, patients with CC deformity performed poorer on SIR. Patients with CC deformity frequently have decreased spiral ganglion cells of the cochlear nerve, resulting in reduced performance on speech perception [20, 31]. It could be hypothesized that this decreased auditory performance may then translate to a decreased overall capability of speech perception. However, because only three studies were included that reported SIR results, it is difficult to conclude whether IEM significantly impacts SIR.

### Operative outcomes—CSF gusher

In conjunction with speech perception and production, operative outcomes are crucial to prepare for and expect during surgery. CSF oozing and gushers occurred most in patients with IP-II and IP-III malformation, EVA syndrome, or a combination of the two [2, 21, 32]. Benchetrit et al. deduced that the increased incidence of CSF gusher in patients with IP-II and EVA syndrome is likely due to direct communication between CSF and the inner ear [33]. However, all cases of gushers were conservatively managed and controlled by either soft tissue packing, reverse Trendelenburg positioning during surgery, or lumbar drainage in more challenging cases [34–36]. Bayazit found that revision due to recurrence of CSF leakage was rare, only occurring among patients with EVA and IP-1 [2]. Choi et al. found that all patients with IP-III had CSF gusher, highlighting the increased risk due to the absence of the modiolus resulting in direct communication between the cochlear base and the internal auditory canal [21]. These results emphasize that for patients with IEMs undergoing cochlear implantation, even the most severe intraoperative risks can usually be managed conservatively, reserving invasive surgical intervention for rare, severe cases. Preoperative imaging and meticulous surgical planning are crucial to reducing the risk of gusher occurrence intraoperatively.

### Operative outcomes—facial nerve stimulation

Postoperative facial nerve stimulation was more prominent in patients with CC deformity with an incidence of greater than 50% [23]. The aberrant positioning of the facial nerve and combined cochleovestibular structure in patients with CC deformities increases the risk of facial nerve stimulation following cochlear implantation. As described by Sennaroğlu, the facial nerve migration during embryogenesis may be abnormal if the basal turn of the cochlea is not properly formed, as is the case with CC deformity [6]. Patients with IP (I, II, or III) had the lowest rate of aberrant postoperative facial nerve stimulation [37, 38]. Facial nerve aberrations require detailed surgical planning as well as anatomical mapping with surgical modifications to ensure a successful cochlear implantation in patients with IEMs [39]. Surgical intervention in these patients may further require alternative approaches such as retrofacial approach, transaditus approach, transcanal approach, or a combined transmeatal approach with posterior tympanotomy [35, 39–41]. Most cases of aberrant facial nerve stimulation were controlled by turning off select electrodes, decreasing the C-level, or readjusting the electrode map, as discussed in the study of Naito et al. [42].

### Operative outcomes incomplete insertion

Adunka et al. concluded that incomplete insertion of the electrode array was most common in patients with IEM [43]. Of the 121 patients whose insertion rate was discussed within the included studies, 6 patients (4.9%) had incomplete insertion, which is larger than reported by the study of Farhood et al. Anatomy that shortens the length of the cochlea (such as cochlear hypoplasia) or makes the cochlea difficult to navigate should be considered, depending on the severity of the hypoplasia, when deciding the size of the electrode to use for cochlear implantation. Given the increased risk of insertion of the electrode array into the vestibule or internal auditory canal in IEM patients, modified surgical approaches may be needed, especially in cases of difficult cases such as IP-III in which risk of erroneous insertion into the internal auditory canal is high [44]. Electrodes must be chosen based on preoperative imaging to decrease the risk of electrode failure or operative complications [45]. In IP-III patients, specifically, there is a higher risk of insertion into the internal auditory canal. A recently published study by Minami et al. categorized IEMs based on the severity of modiolus deficiency and internal auditory canal/cochlear nerve deficiency to help address the types of electrodes that could be used for particular IEMs [46]. This study suggests that a perimodiolar electrode should not be utilized in patients with IP-I, IP-III, or common cavity, due to cochlear dysplasia or modiolus defects. However, in patients where the modiolus is present, but the cochlear nerve is deficient, such as IAC stenosis, cochlear aperture stenosis, or IP-II, a perimodiolar electrode can provide increased intensity to perpetuate signals through a deficient cochlear nerve [46]. Historically, a one-size-fits-all method has been used, however, electrodes have undergone multiple technological advancements over the past 30 years. Newer electrodes utilize various lengths and orientations as well as advanced processing systems and software, permitting the clinicians to tailor the electrode towards specific patient anatomy [47].

### Limitations

There is very little standardization for the timing and administration technique of speech perception tests in CI [48]. In general, closed-set tests are easier for patients to take as their answer choices are limited to the choices provided to them, while open-set tests are more difficult [49]. Few studies included in this review did not separate open-set tests from closed-set tests, making deduction of speech perception issues difficult to pinpoint.

Interpretation and comparison of results was also complicated by varying length of data collection and follow-up. For example, several studies collected data over 15–20 years, during which time surgical techniques and technology have improved, making it difficult to directly compare patients at the beginning of a study to those at the end. Appropriate comparison of cochlear implantation outcomes in patients with IEMs against patients with normal anatomy may also provide better insight into the expectations that need to be discussed with parents of patients with IEMs. However, given the rarity of IEMs, it is difficult to fault studies for gathering data in this method. In addition, to ensure inclusion of studies with comparable controls, many chart reviews and case series studies with strong data were not included. Also, given the commonality of IEM with concurrent neurological disorders, it would be interesting in future studies to evaluate the effect of additional handicap (autism spectrum disorders and pervasive developmental disorders) on audiometric outcomes [2].

Although rare, post-operative infection, including meningitis, can have significant and devastating long-term effects on cochlear implant outcomes. As meningitis has been shown to be more common among patients with IEM, there is a need to identify infection sources as well as tailor the post-operative treatment course in patients with IEM [50]. To limit cases of meningitis, it has been suggested to encourage vaccination of patients prior to surgery and have shorter follow-up intervals in patients who experienced perilymph gushers, perilymph oozers, otorrhea, or rhinorrhea [50].

Furthermore, this review was limited to studies searchable through PubMed (MEDLINE), Science Direct, Web of Science, Scopus, and EMBASE databases and mapped to the MeSH search terms commonly used in systematic reviews, which may have impacted the results reported.

## Conclusions and future directions

The results of this systematic review suggest that cochlear implantation is an appropriate intervention in patients with IEMs. In many cases, improvements in audiometric outcomes, including speech perception and production following cochlear implantation in patients with IEM, were equivalent to those without IEMs. However, the more extensive IEMs such as CC deformity and severe cochlear dysplasia, while reported to have had improvements from baseline, generally had significantly poorer outcomes compared to matched controls. Intraoperative and postoperative complications such as CSF gushers and facial nerve stimulation are not uncommon in this patient population. CSF gushers can often be managed conservatively. Incomplete insertion of the electrode during cochlear implantation in these patients raises another concern. Therefore, careful planning prior to implantation can help improve outcomes when considering the type of malformation and the cochlea structures involved. The recommendation of cochlear implantation for most patients with IEMs is reasonable given the improvements in audiometric outcomes and the appropriate perioperative evaluation to detect potential surgical complications. However, it is important to manage family expectations, particularly when considering patients with more extensive deformities.

Further large-scale, longitudinal cohort studies with matched control groups are warranted, which will better inform us regarding the clinical outcomes of cochlear implantation in individuals with IEMs. It is important to note, however, that patients with cochlear nerve abnormalities should expect decreased improvement. There are currently no standardized guidelines regarding whether patients with IEMs should undergo cochlear implantation. Thus far, most clinicians must rely on their clinical judgment and evidence to decide whether patients would benefit from CIs. This paper hopes to provide insight regarding the risks and benefits of cochlear implantation in patients with IEMs.

## Supporting information

S1 Checklist PRISMA 2020 checklist. (PDF)
